# Metabolic changes and anti-tumor effects of a ketogenic diet combined with anti-angiogenic therapy in a glioblastoma mouse model

**DOI:** 10.1038/s41598-020-79465-x

**Published:** 2021-01-08

**Authors:** Masahiro Maeyama, Kazuhiro Tanaka, Masamitsu Nishihara, Yasuhiro Irino, Masakazu Shinohara, Hiroaki Nagashima, Hirotomo Tanaka, Satoshi Nakamizo, Mitsuru Hashiguchi, Yuichi Fujita, Masaaki Kohta, Eiji Kohmura, Takashi Sasayama

**Affiliations:** 1 Department of Neurosurgery, Kobe University Graduate School of Medicine, 7-5-1, Kusunoki-cho, Chuo-ku, Kobe, 650-0017 Japan; 2 Department of Neurosurgery, Nishi-Kobe Medical Center, Kobe, Japan; 3 Division of Evidence-Based Laboratory Medicine, Kobe University Graduate School of Medicine, Kobe, Japan; 4 Integrated Center for Mass Spectrometry, Kobe University Graduate School of Medicine, Kobe, Japan; 5 Division of Epidemiology, Kobe University Graduate School of Medicine, Kobe, Japan; 6 Department of Neurosurgery, Massachusetts General Hospital Research Institute, Boston, MA USA

**Keywords:** Cancer, Oncology

## Abstract

The ketogenic diet (KD) is a high fat and low carbohydrate diet that produces ketone bodies through imitation of starvation. The combination of KD and Bevacizumab (Bev), a VEGF inhibitor, is considered to further reduce the supply of glucose to the tumor. The metabolite changes in U87 glioblastoma mouse models treated with KD and/or Bev were examined using gas chromatography-mass spectrometry. The combination therapy of KD and Bev showed a decrease in the rate of tumor growth and an increase in the survival time of mice, although KD alone did not have survival benefit. In the metabolome analysis, the pattern of changes for most amino acids are similar between tumor and brain tissues, however, some amino acids such as aspartic acid and glutamic acid were different between tumors and brain tissues. The KD enhanced the anti-tumor efficacy of Bev in a glioblastoma intracranial implantation mouse model, based on lowest levels of microvascular density (CD31) and cellular proliferation markers (Ki-67 and CCND1) in KD + Bev tumors compared to the other groups. These results suggested that KD combined with Bev may be a useful treatment strategy for patients with GBM.

## Introduction

Glioblastoma (GBM) is a very poor prognosis brain tumor, with a median survival of 16 months after treatment with temozolomide (TMZ), bevacizumab (Bev), a vascular endothelial growth factor (VEGF) inhibitor, and radiation^1^. Several new treatments have been developed, but the prognosis of GBM remains poor^2,3^. Therefore, developing new treatment methods is urgent. Tumor cells take in a large amount of glucose and synthesize not only ATPs but also nucleic acids and fatty acids for cell maintenance^4^. Different from normal cells, the glycolytic pathway develops in an aerobic state and takes a metabolic form that is suppressed to the TCA cycle and electron transport system^4^. This is called the Warburg effect^5^. Among the treatments that take into account the Warburg effect, ketogenic diet (KD) therapy is emerging as a viable complementary therapeutic strategy for GBMs.

KD is a high fat and low carbohydrate diet that produces ketone bodies through imitation of starvation. In 1921, KD was developed to treat epilepsy^6^. KD induces ketone bodies that are transported to many organs including brain, metabolized in the mitochondria, and used as an energy source instead of glucose. Especially for the brain, it is the only energy source when glucose is depleted. However, since tumor cells depend on glucose as an energy source, it is thought that ketone bodies cannot be used. Glioma cells are reported to be incapable of compensating for glucose restriction by metabolizing ketone bodies^7,8^. Due to the "metabolic inflexibility" of glioma, KD is attracting attention as an adjuvant treatment for glioma.

There are reports on the anti-tumor effect of KD on tumor cells, but its efficacy has not yet been clarified. Zhou and Seyfried reported that an intracerebral glioma mouse model had longer survival under a calorie-restricted KD^9,10^. And Poff et al. reported KD decreased blood glucose, slowed tumor growth, and increased mean survival time in mice with systemic metastatic cancer^11^. Feyter et al. found no anti-tumor effect of KD on an RG2 and 9L glioma mouse model with the increase of transport and oxidation of ketone bodies^12^.

GBM is a highly vascularized brain tumor and an attractive target for anti-angiogenic therapies^13^. VEGF has been identified as a critical regulator of angiogenesis, and VEGF inhibitors are used in the treatment of newly or recurrent GBMs. Administration of bevacizumab, a VEGF inhibitor, significantly decreases global cerebral blood flow (CBF) with a potentially preferential decrease in tumor perfusion compared with normal brain tissue^14^. Keunen et al. reported that anti-angiogenic treatment leads to major vessel remodeling, resulting in reduced perfusion and an increase in hypoxia in the tumor microenvironment^15^. Since VEGF inhibitors reduce the blood flow of tumors and promote ischemic conditions, using VEGF inhibitors in combination with a low-glucose KD further deprives glucose supply. Therefore, we analyzed the anti-tumor effect of combination therapy of KD and VEGF inhibitor.

## Results

### Survival benefit by the combination of ketogenic diet and bevacizumab

**Figure 1:**
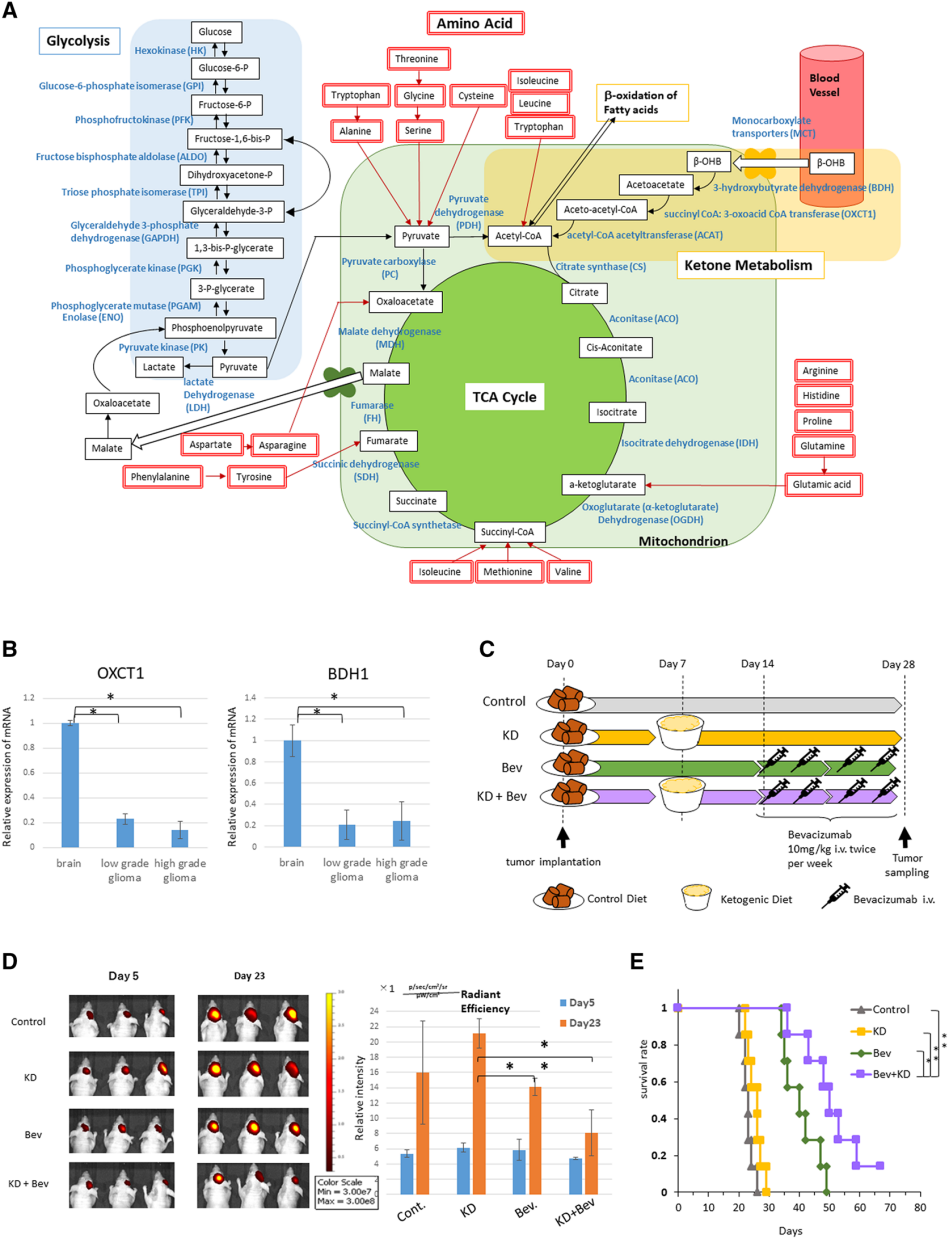
(**A**) Metabolic pathway of glucose, ketone body, and amino acid. (**B**) Comparison of mRNA expression levels of ketone metabolizing enzymes (OXCT1 and BDH1) in peripheral normal brain (n = 2), low grade gliomas (n = 4) and high grade gliomas (n = 12) (*p < 0.05, **p < 0.01). (**C**) Treatment schedule for mice. The ketogenic diet is started 7 days after transplantation, and bevacizumab treatment is started 14 days after transplantation. Bevacizumab is administered at a dose of 10 mg/kg intravenously via the tail vein. The implanted mice are divided into 4 groups: standard diet (control), ketogenic diet alone (KD), bevacizumab alone (Bev), and ketogenic diet plus bevacizumab (KD + Bev). Five mice/group in metabolome analysis, 5 mice/group in microarray analysis, 7 mice/group in survival analysis, and 3 mice/group in in vivo image analysis were examined. One month later, the mice are sacrificed and tumors are collected, and metabolome analysis, histological analysis, and microarray analysis are conducted. (**D**) Comparison of the tumor growth in each treatment group. U87 IVIS cells are transplanted into the mice brain and treat with a ketone diet and bevacizumab. Fluorescence is measured 6 days and 23 days after transplantation. After 23 days, the fluorescence intensity of the KD + Bev group is significantly lower than that of the KD group. (**E**) Comparison of the survival curves of the mice in each treatment group. The KD + Bev group has significantly longer survival than any other group (control group, KD group: **p < 0.01, Bev group: *p = 0.025).

First, we examined the expression of the enzymes related to ketone metabolism in the brain and glioma (Fig. 1A). mRNA expression of OXCT1 and BDH1 was significantly lower in gliomas compared to normal brain (Fig. 1B). Immunostaining revealed a very low expression in GBM (Supplementary Fig. S1A). These results were consistent with previous results^8^. Next, U87 cells were transplanted into the brains of mice and the effects of ketogenic diet were examined. Mice treated with the ketogenic diet did not significantly prolong survival compared to mice treated with the normal diet (Supplementary Fig. S1B). The VEGF inhibitor is known to reduce tumor blood supply^15^, therefore, we examined whether the combination of ketogenic diet and Bev therapy had an antitumor effect. The U87 cell implanted mice were divided into four treatment groups (Fig. 1C). In total, experiments were performed on 20 mice in each group. There was no significant difference in body weight and blood glucose levels between them (Supplementary Fig. S1C). However, the blood ketone level was markedly elevated in the KD and KD + Bev group, confirming that ketone was produced in the body (Supplementary Fig. S1C right). In the tumor growth, the KD alone did not show an anti-tumor effect compared to the control, however, the KD + Bev showed a significant decrease compared to the KD alone and a tendency to decrease compared to the control and Bev alone (Fig. 1D). In addition, the survival time of KD + Bev was significantly prolonged compared to all other groups (vs control, KD: p < 0.01, vs Bev: p = 0.025) (Fig. 1E). These results show that the combination of KD and Bev have significant anti-tumor effect and survival benefit.

### Metabolome analysis by GC–MS

**Figure 2:**
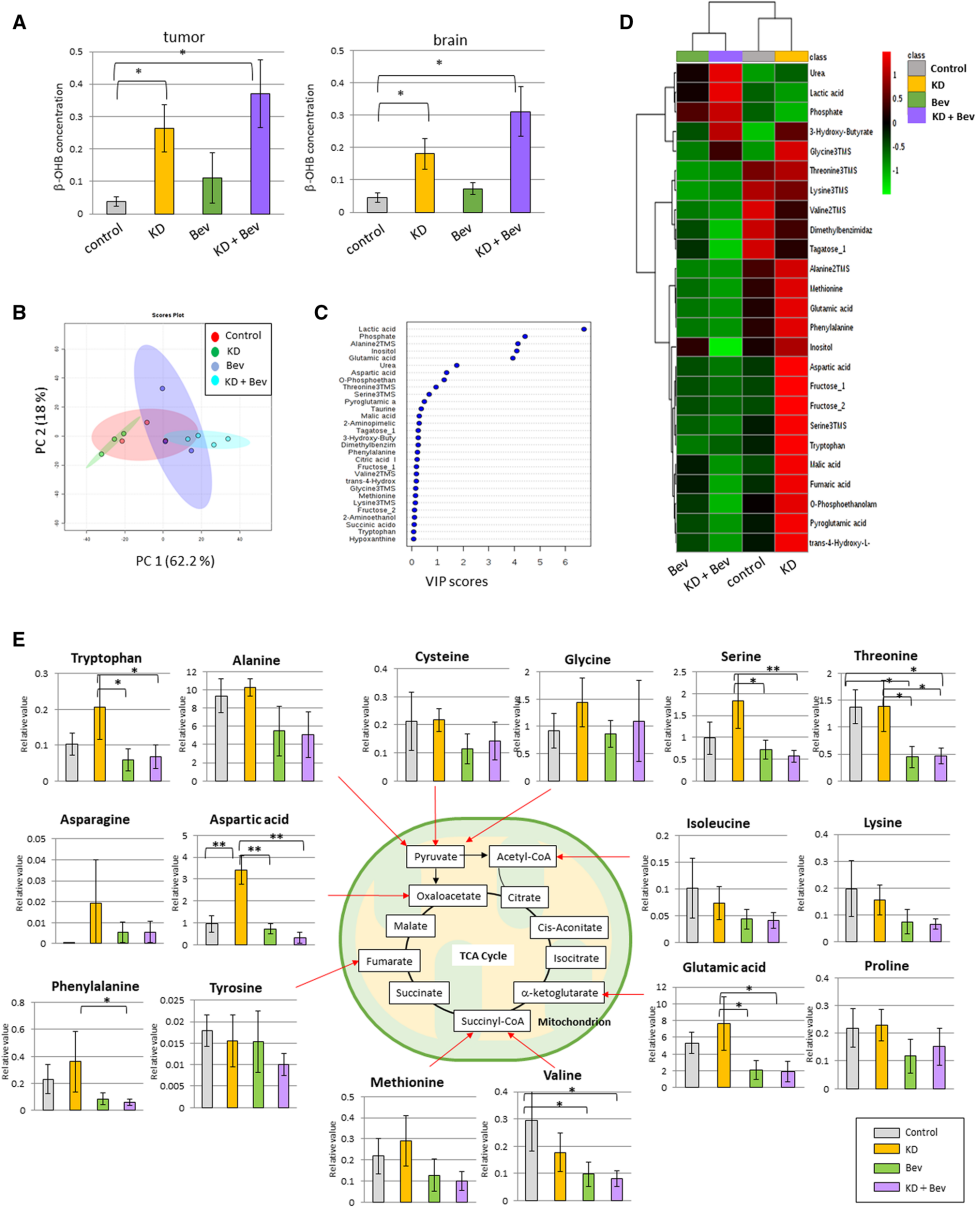
(**A**) Comparison of β-OHB concentration in the tumor and normal brain in each group. In the ketogenic diet groups (KD and KD + Bev), it is markedly increased (*p < 0.05, **p < 0.01, and Tukey–Kramer test). (**B**) PCA analysis of the tumor tissue in each of the four groups. (**C**) VIP score analysis of the tumor tissue. (**D**) Heat map showing characteristic metabolite changes in the tumor tissue in each group. (**E**) Comparison of the amino acids levels in the control, KD, Bev, and KD + Bev groups. Note several amino acids increase by the KD, and most of the amino acids decrease in KD + Bev group. In particular, the change in aspartic acid is most significant (*p < 0.05, **p < 0.01, and Tukey–Kramer test).

The metabolome data are shown in Supplementary Tables S1 and S2. The concentration of β-OHB was significantly increased in both tumors and normal brain in the KD and KD + Bev groups (Fig. 2A). In the principal components analysis, the KD and KD + Bev groups were clearly separated (Fig. 2B). In the VIP score, lactic acid, phosphate, alanine, inositol, glutamic acid, and urea were extracted as metabolites that were involved in the separation (Fig. 2C). In the heat map of metabolome analysis, the 4 groups showed characteristic patterns (Fig. 2D). These results suggested that marked metabolic changes occurred in the KD + Bev group.

### Metabolic changes in the amino acids by ketogenic diet and bevacizumab

In tumor tissues, several amino acids increased in KD and decreased in KD + Bev (Fig. 2E). Aspartic acid increased markedly in the KD group compared to the control, and decreased significantly in the Bev and KD + Bev groups. The KD + Bev group had significantly lower glutamic acid, phenylalanine, serine, threonine, and tryptophan than the KD group (Fig. 2E). Surprisingly, there were no amino acids that were significantly different between the Bev group and the KD + Bev group. In the normal brain, the pattern of changes for most amino acids are similar between tumor and brain tissues. For example, the KD increased the levels of tryptophan, phenylalanine, tyrosine and proline in tumor tissues as well as brain tissues and so on (Supplementary Fig. S2) However, some amino acids were different in tumors and brain tissue. The amino acids that differed most markedly between brain and tumor were aspartic acid and glutamic acid. On the other hand, cysteine, glycine, isoleucine, lysine, proline, and tyrosine of the brain were almost the same changes as the tumor. These results suggest that aspartic acid and glutamic acid may be important metabolites of the tumor under the KD.

### Changes of metabolites and mRNA expression of the TCA cycle and glycolysis

**Figure 3:**
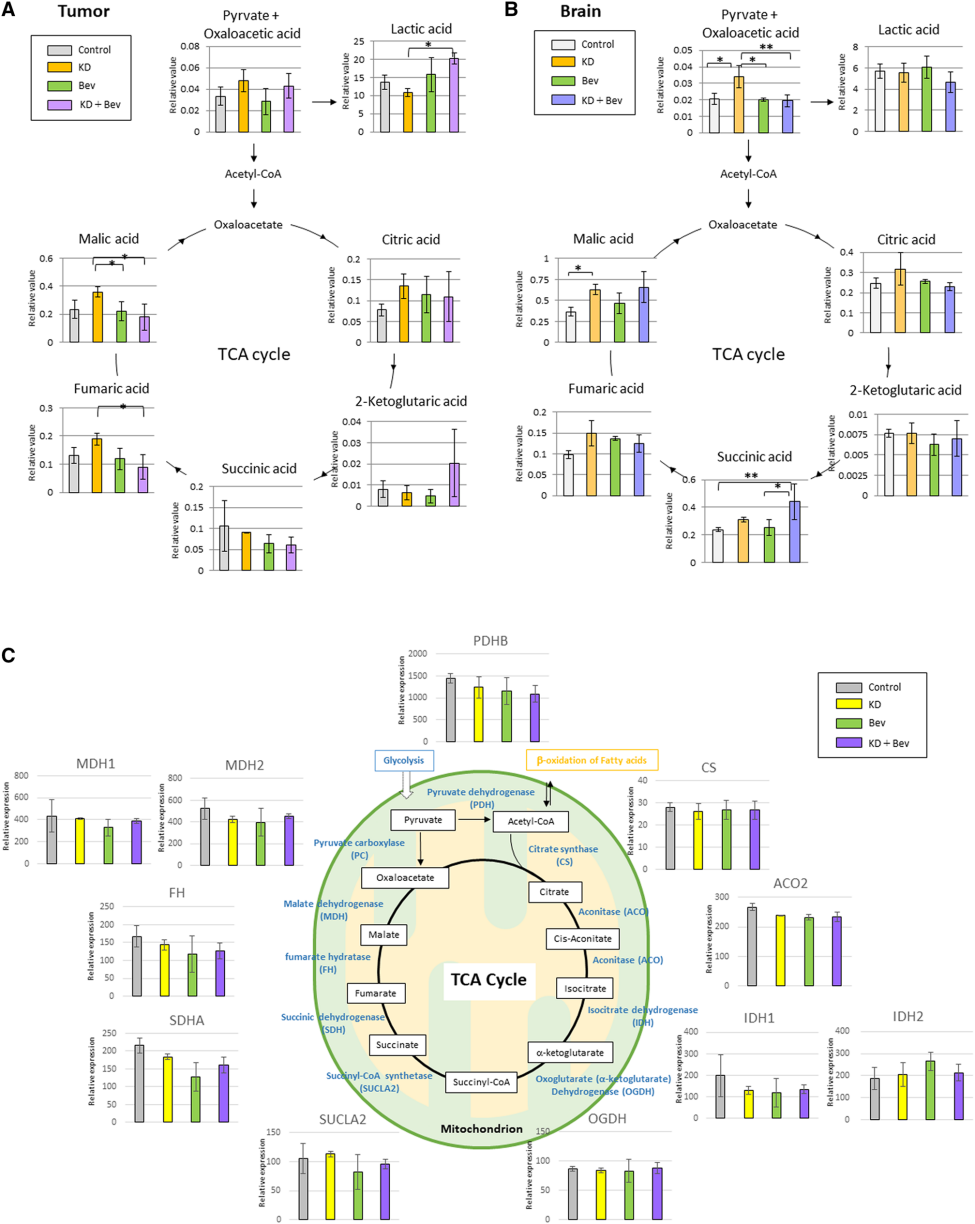
(**A**) Metabolome analysis of TCA cycle-related metabolites in the tumor tissue. (*p < 0.05, **p < 0.01, and Tukey–Kramer test) (**B**) Metabolome analysis of TCA cycle-related metabolites in the normal brain tissue. (*p < 0.05, **p < 0.01, and Tukey–Kramer test) (**C**) Comparison of mRNA expression levels of TCA cycle-related enzymes. (*p < 0.05, **p < 0.01, and Tukey–Kramer test).

In the tumor tissue, the lactic acid of KD + Bev was significantly increased and fumaric acid and malic acid of KD + Bev were significantly decreases compared with KD (Fig. 3A) In addition, 2-ketoglutartic acid tended to increase in the KD + Bev group. On the other hand, in the normal brain, there was no significant difference in lactic acid between the groups (Fig. 3B). In addition, there were no difference of fumaric acid and malic acid between KD and KD + Bev. However, malic acid and pyruvate + oxaloacetic acid of KD were significantly increased compared with control, and succinic acid of KD + Bev was significantly increased compared with control (Fig. 3B). We examined the mRNA expression levels of the TCA cycle and glycolysis-related enzymes in tumor using the microarray analysis (Supple. Table S3). However, there was no difference in TCA cycle between the treatment groups (Fig. 3C). Phosphofructokinase-muscle (PFKM) were significantly decreased in the KD + Bev group compared with control (Supplementary Fig. S3). The level of ENO1 was significantly reduced in all groups KD, Bev and KD + Bev compared to control group (Supplementary Fig. S3).

### Changes in the expression level of ketone metabolism-related enzymes

**Figure 4:**
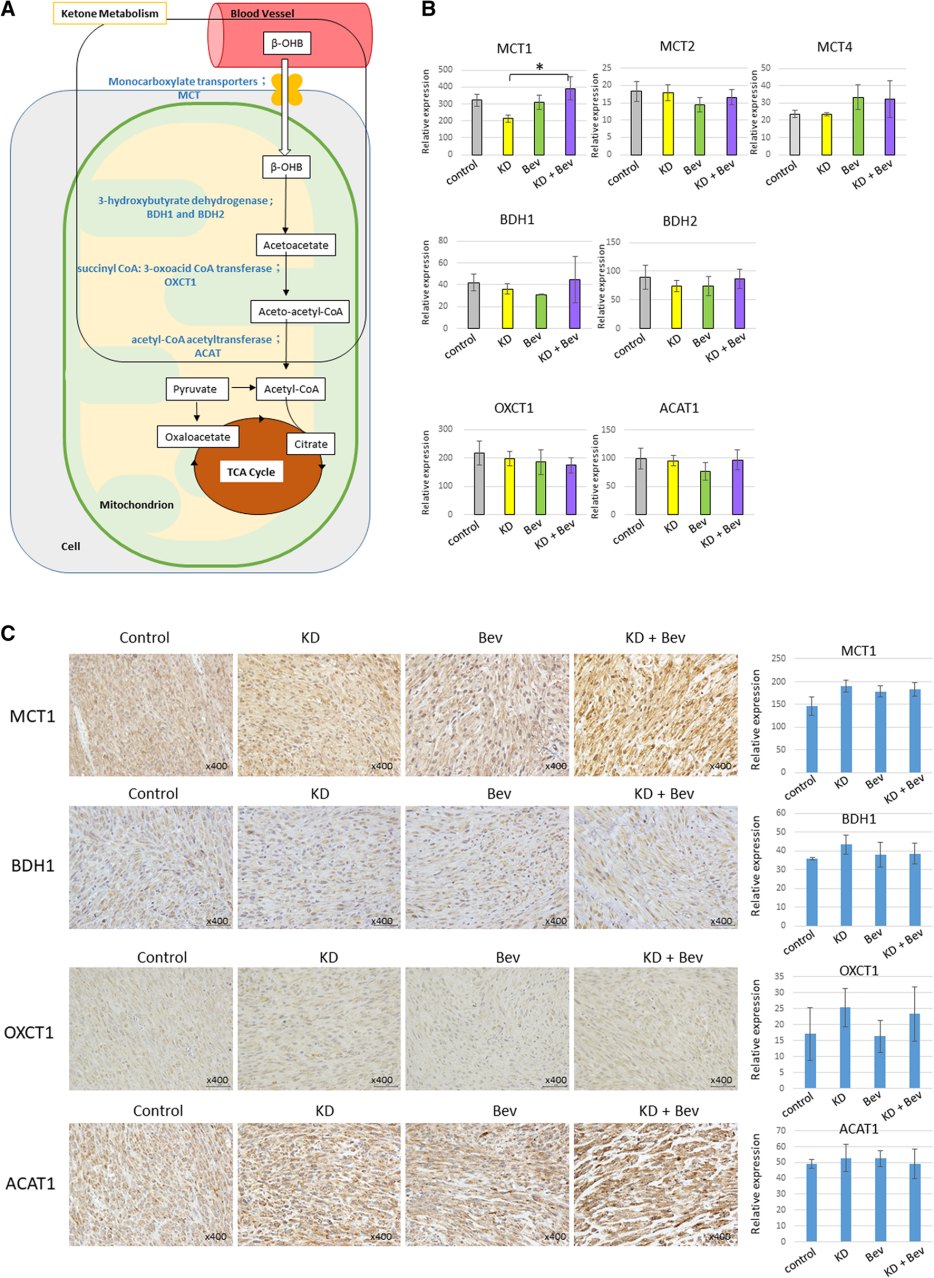
(**A**) Schema of the ketone metabolic pathway. (**B**) Comparison of mRNA expression levels of ketone metabolizing enzymes or ketone body transport proteins. (*p < 0.05, **p < 0.01, and Tukey–Kramer test). (**C**) Immunostaining of ketone metabolizing enzymes (OXCT1, BDH1, and ACAT1) and ketone body transport protein (MCT1). Immunostaining analyses analyze 5 tumors in each group. An intensity of immunostainings is quantified using image J software.

The mRNA expression of BDH1, BDH2, OXCT1, and ACAT1 in tumor did not differ between the treatment groups (Fig. 4A,B). Among the ketone body transporter proteins, MCT1 showed an increase in the KD + Bev group (Fig. 4B). But, there was no difference between each group in immunohistochemical experiment (Fig. 4C). The expressions of BDH1, OXCT1 and ACAT1 were almost the same in the four groups (Fig. 4C).

### Anti-proliferation effects of ketogenic diet and bevacizumab

**Figure 5:**
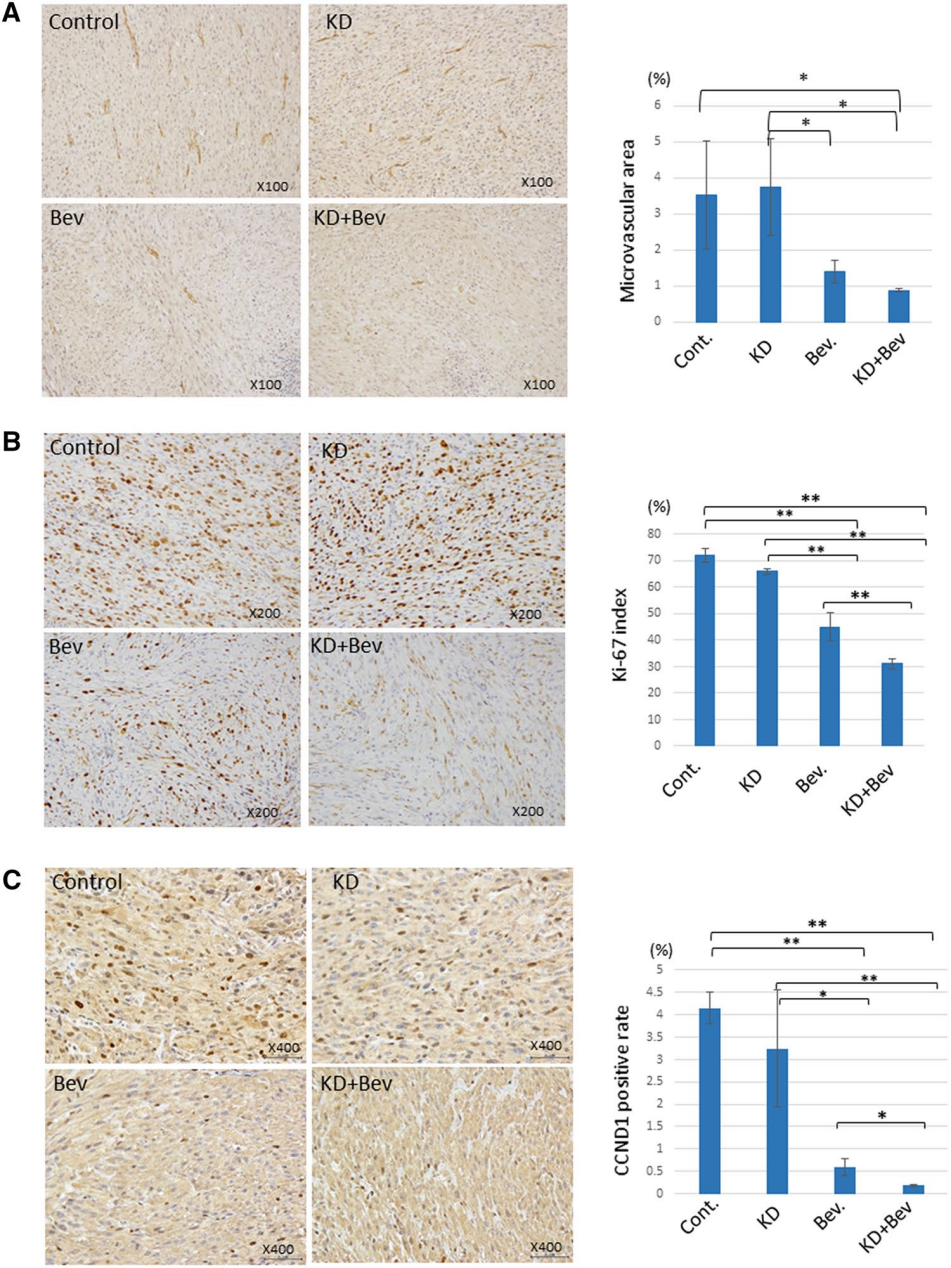
(**A**) Comparison of the vascular density in the tumor tissue in each treatment group. Tumor tissue is immunostained with CD31 to quantify the vascular area. Immunostaining analyses analyze 5 tumors in each group. In the KD + Bev group, the blood vessel density is significantly lower than in the control and KD groups. (*p < 0.05, **p < 0.01, and Tukey–Kramer test) (**B**) Comparison of Ki-67 immunostaining and Ki-67 index of the tumor tissue in each treatment group. Immunostaining analyses analyze 5 tumors in each group. The Ki-67 index is significantly lower in the KD + Bev group than in any other group. (*p < 0.05, **p < 0.01, and Tukey–Kramer test) (**C**) Comparison of tumor tissue by cyclin D1 immunostaining. Immunostaining analyses analyze 5 tumors in each group. In the control and KD group, cyclin D1 is markedly accumulated in the nucleus, but in the KD + Bev group, the accumulation in the nucleus is significantly reduced. (*p < 0.05, **p < 0.01, and Tukey–Kramer test).

The Bev and KD + Bev group showed a significant decrease of microvascular density, suggesting that administration of Bev markedly suppressed angiogenesis (Fig. 5A). In Ki-67 staining, the proportion of proliferating cells was significantly reduced in the KD + Bev group (Fig. 5B). In addition, the accumulation of cyclin D1 in the nucleus was markedly reduced in the KD + Bev group (Fig. 5C). These results confirmed that the combination of the KD and Bev therapy suppressed the proliferation of tumor cells.

## Discussion

The effects of KD on GBM are controversial^12^. However, many reports demonstrated the effect of calorie restricted KD therapy^9^, suggesting that stronger starvation is necessary. Therefore, VEGF inhibitors that render tumors ischemic may be useful in promoting the anti-tumor effects of KD. In this study, we showed the utility of a combination of KD and anti-VEGF therapy in a mouse model. Bevacizumab is a drug used for the standard treatment of GBM. It suppresses angiogenesis and blood flow in tumors and normalizes tumor blood vessels^14–16^. Bevacizumab had no effect on survival with radiation-temozolomide therapy, the basic GBM treatment. The reason for this is unknown, but it appears that bevacizumab has no cytotoxic effect. However, when bevacizumab is administered, contrast enhancement in the tumor is reduced and calcification is often caused, so dynamic changes occur in the tumor. Rieger et al. conducted a pilot study of KD in patients with recurrent glioblastoma, and the tumor shrinkage was good with the concomitant use of bevacizumab^17^.

To the best of our knowledge, there are no reports of metabolome analysis in the brain and tumors following KD plus Bev therapy. Artzi et al. showed in proton magnetic-resonance-spectroscopy (1H-MRS) of patients with low-grade glioma, the levels glutamine and glutamate in tumor were increased after treatment with KD alone^18^. In our metabolome analysis, we observed reduction of threonine, valine, isoleucine, leucine levels in glioma tissue of the groups Bev and KD + Bev. Similarly, Aminzadeh-Gohari et al. also reported decreased level of most essential amino acids (EAAs) in plasma and neuroblastoma tumor tissues upon the treatment of the KD in combination with chemotherapy^19^. Furthermore, the combination of the KD and chemotherapy also reduced tumor blood-vessel density and intratumoral hemorrhage in neuroblastoma xenografts^19^. There are a total of nine amino acids (histidine, isoleucine, leucine, lysine, methionine, phenylalanine, threonine, tryptophan, and valine) that are essential in human bodies. Compared with their normal tissues, tumor cells frequently exhibit an increased demand for EAAs. Yue et al. showed oncogenic MYC promotes SLC7A5/SLC43A1-mediated essential amino acid (EAA) uptake^20^.

On the other hand, KD showed a significant increase in the levels of tryptophan, phenylalanine, pyruvate + oxaloacetate and malic acid in normal brain tissue compared to the control diet. These showed similar changes in glioma tissue. Interestingly, however, aspartic acid was significantly elevated by KD in glioma tissue, but not in normal brain tissue at all. In addition, aspartic acid, which is significantly increased by KD in glioma tissues, was not increased by KD + Bev. Aspartic acid, one of the non-EAAs, plays an important role in the TCA cycle, ornithine cycle, and biosynthesis of nucleic acids^21^. Aspartic acid is a precursor for asparagine, isoleucine, methionine, lysine, threonine, pyrimidines, NAD, and pantothenate; a nitrogen donor for arginine and purine synthesis; and an important metabolic effector^22^. Aspartic acid is easily converted into oxaloacetate and is used for gluconeogenesis. In addition, aspartic acid is not only important as a component of the urea cycle, but also involved in the entry and exit of substances from mitochondria (malic acid-aspartate shuttle). Mitochondrial aspartate export is essential for de novo nucleotide synthesis^23^.

There are few studies of metabolome analysis of tumors after anti-VEGF therapy. The study conducted by Fack et al. showed metabolites associated with the TCA cycle were significantly reduced in both the tumor and brain when bevacizumab was administered^24^. However, in our experiments, treatment with bevacizumab alone did not show a significant reduction in TCA cycle-related metabolites. These differences may have been due to variations in the transplanted cells.

There are several reports of other treatments used in combination with KD in glioma therapy. In a study of the combination of KD and radiation therapy, the combination therapy significantly increased the survival time of mice^25^. There are also reports that anti-tumor effects have been observed with the combined use of 2-deoxy-d-glucose (2-DG) and hyperbaric oxygen therapy^11,26^. It was recently reported that a combination of calorie-restricted KD and glutamine-targeted therapy was effective^27^. There are several human clinical studies in KD, but high-quality evidence on the effect of KD on tumor and QoL is currently lacking in glioma patients. Martin-McGill et al. conducted a randomized pilot study to investigate the use of KD as an adjuvant therapy for patients with GBM (NCT03075514). Woodhouse et al. showed that modified Atkins diet, less stringent version of KD, was feasible and safe for glioma patients during radiation and chemotherapy treatment. Several clinical studies of KD in combination with radiation and temozolomide (NCT03278249, NCT03451799) are ongoing. however, there is no study of KD in combination with Bev. From our result, clinical study of KD plus Bev is necessary to evaluate the effect of glioblastoma patients.

There are several limitations to this study. Only one type of U87 cell was analyzed. Although U87 cells are widely used in glioblastoma research, the metabolism of tumors varies. Research using other glioblastoma cells may be necessary in the future. Second, the metabolome analysis used the GC–MS only and the number of metabolites that was identified was limited. A combination of metabolome analysis, such as liquid chromatography-mass spectrometry (LC–MS), may elucidate in more detail the metabolic changes in KD + Bev therapy.

In conclusion, the KD enhanced the anti-tumor efficacy of Bev in a glioblastoma intracranial implantation mouse model, based on lowest levels of cellular proliferation markers (Ki67 and CCND1) in Beb + KD-tumors compared to the other groups. Since both anti-angiogenic therapy and KD are used in clinical settings and their safety has been confirmed, combination therapy is feasible. Clinical trials using this combination should be conducted in the future.

## Materials and methods

### Cells and reagents

U87 malignant glioma cells obtained from the American Type Culture Collection and U87 cells expressing iRFP720 cell lines obtained from Mischel PS. KetoCal for ketogenic diet was obtained from Nutricia North America (Nutricia North America, Gaithersburg, MD), and bevacizumab was obtained from Chugai Pharmaceutical Co., Ltd. (Chugai Pharmaceutical Co., Ltd., Tokyo, Japan) Antibodies to ACAT1, CD31, Ki-67, and cyclin D1 were purchased from Abcam (Abcam, Cambridge, MA, USA). OXCT1 and BDH1 was purchased from Atlas Antibodies (Atlas Antibodies, Bromma, Sweden). MCT1 was purchased from Santa Cruz Biotechnology (Dallas, TX, USA).

### Clinical specimens

Glioma and peripheral normal brain tissues were obtained from the patients operated at the Department of Neurosurgery, University of Kobe. This study was performed in accordance with Good Clinical Practice and the Declaration of Helsinki. All methods were carried out in accordance with the relevant directives and regulations, and informed consent was obtained from all the participants for the experiments. This study was approved by the ethical review boards of Kobe University Clinical Research Ethical Committee (No. 1714).

### Real-time PCR of glioma samples

For BDH1 and OXCT1 expression analysis, 16 glioma samples (4 low grade gliomas and 12 high grade glioma) and 2 peripheral normal brain tissues were used. Total RNA was obtained using a mirVana miRNA Isolation Kit. Reverse transcription was done with a High Capacity cDNA Reverse Transcription Kit (Applied Biosystems), according to the manufacturer’s instructions. mRNA expression was analyzed by the ΔΔ-Ct method using TaqMan Gene Expression Assays (The Thermo Fisher Scientific, Inc.), according to the manufacturer’s instructions. This study was approved by the ethical review board of our institutions (No. 1714).

### In vivo experiments

Eight-week-old male nude mice (BALB/c-nu/nu; Claire, Japan) were used. All mice were bred and kept at Kobe University in accordance with Laboratory Animal Resources Commission standards. All experiments were approved by the Institutional Animal Care and Use Committees of Kobe University (P160805-R2). The mice were stratified into 4 groups: control, ketogenic diet (KD), bevacizumab (Bev), and ketogenic diet plus bevacizumab (KD + Bev). The control and Bev groups were fed a standard diet (CLEA Rodent Diet CL-2) (CLEA Japan, Inc., Tokyo, Japan) ad libitum. The KD and KD + Bev groups were fed a KD of KetoCal 4:1 (Nutricia, Hoofddorp, Netherlands) ad libitum 7 days after transplantation. The dietary components were showed in Supplementary Table S4. KD paste (2:1 mix of KetoCal and water) was provided to the animals daily. Two weeks after transplantation, the Bev and KD + Bev groups received intravenous injections of bevacizumab (10 mg/kg) in the tail vein twice a week for 2 weeks. Four weeks after transplantation, the mice were sacrificed, their brains were removed, and the tumors were collected. In intracranial implantation, the mice were anesthetized to sedate and placed in a stereotactic apparatus (SP-6 model, Narimo Scientific Research Institute, Inc.). A burr hole was drilled 2.0 mm to the right of the bregma. A needle was inserted to a depth of 3.5 mm from the surface of the brain. Then 10 μl of U87 cells (10^6^ cells) were infused. Body weight was measured twice a week. Blood glucose and blood ketone levels were measured with a dedicated measuring instrument (FreeStyle Optium Neo; Abbott, Japan). In survival analysis, seven mice of each group were analyzed. In tumor growth analysis using in vivo imaging, three mice of each group were analyzed. In metabolome analysis and microarray analysis, 5 mice of each group were analyzed.

### GC–MS analysis

GC–MS analysis of the collected samples was conducted using a GCMS-QP2010 Ultra (Shimadzu Co., Kyoto, Japan) according to a previous report^28^. A fused silica capillary column (CP-SIL 8 CB low bleed/MS; 30 m × 0.25 mm inner diameter, film thickness: 0.25 µm; Agilent Co., Palo Alto, CA, USA) was used for this analysis. The column temperature was maintained at 80 °C for 2 min and then increased at 15 °C/min to 330 °C and maintained for 6 min. The transfer line and ion source temperatures were 250 °C and 200 °C, respectively. Twenty scans per second were recorded over a mass range of 85–500 *m/z* using the Advanced Scanning Speed Protocol (ASSP, Shimadzu Co.). Data processing was conducted as described in a previous report^29^. The MS data were exported in netCDF format. Peak detection and alignment were conducted using MetAlign software (Wageningen UR, the Netherlands). The resulting data were exported in CSV format and analyzed using in-house analytical software (AIoutput)^30^. This software enables peak identification and semi-quantification using an in-house metabolite library. For the semi-quantitative assessment, the peak height of each quantified ion was calculated and normalized using the peak height of 2-isopropylmalic acid as an internal standard.

### DNA microarray analysis

Complementary DNA (cDNA) was synthesized using an Ambion WT Expression Kit (Thermo Fisher Scientific, Waltham, MA, USA) according to the manufacturer’s instructions. Microarray analysis was performed using a 3D-Gene Human Oligo chip 25 K (Toray Industries Inc., Tokyo, Japan). Total RNA was labeled with Cy5 using an Amino Allyl MessageAmp II aRNA Amplification Kit (Applied Biosystems, Foster City, CA, USA). The Cy5-labeled RNA pool was mixed with hybridization buffer and hybridized for 16 h according to the manufacturer’s instructions. A 3D-Gene Scanner (Toray Industries Inc., Tokyo, Japan) and 3D-Gene Extraction software (Toray Industries Inc., Tokyo, Japan) was used for analyses.

### In vivo imaging

In vivo imaging experiments using U87 in which iRFP720 was expressed were performed with 3 mice in each group. Images were obtained twice a week using IVIS Lumina LT in vivo imaging system (PerkinElmer, Inc., Waltham, MA, USA). To confirm the tumor growth tendency, Living Image software (ver.4.5) was used.

### Analysis of microvascular density

Immunohistochemical staining of CD31 was independently evaluated and scored by the neuro-oncologist, K.T., who was blind to the experimental data. The mean percentage of the CD31 immunoreactive area was calculated from five randomly selected fields per section under × 200 middle-power magnification. The images were analyzed using ImageJ 1.48 software (NIH, Bethesda, MD, USA).

### Statistical analysis

The Mann–Whitney U-test was used to analyze the differences between two groups. The statistical significance among three groups or more was determined using the Tukey–Kramer test. Survival was estimated using the Kaplan–Meier method, and the significance was determined using the log-rank test. Overall survival (OS) was defined as the time from the onset of implantation to death. A p-value < 0.05 was considered statistically significant. Statistical analysis was performed using JMP 11 software (JMP Institute, Cary, NC, USA).

## Supplementary Information

Supplementary Information.

Supplementary Table 1.

Supplementary Table 2.

Supplementary Table 3.

Supplementary Table 4.

## Abbreviations

GBM: Glioblastoma
VEGF: Vascular endothelial growth factor
Bev: Bevacizumab
KD: Ketogenic diet
GC–MS: Gas chromatography–mass spectrometry
TCA cycle: Tricarboxylic acid cycle
OXCT1: Succinyl CoA: 3-oxoacid CoA transferase
BDH1: 3-Hydroxybutyrate dehydrogenase 1
ACAT1: Acetyl-Coenzyme A acetyltransferase 1
MCT1: Monocarboxylate transporter 1
