# Supplementary material for: Metabolic changes and anti-tumor effects of a ketogenic diet combined with anti-angiogenic therapy in a glioblastoma mouse model
Source: Sci Rep. 2021 Jan 8;11:79. doi: 10.1038/s41598-020-79465-x (PMC7794443; doi:10.1038/s41598-020-79465-x)
Supplement: Supplementary file 1 — Supplementary Information. [file 41598_2020_79465_MOESM1_ESM.docx]

**Metabolic changes and anti-tumor effects of a ketogenic diet combined with anti-angiogenic therapy in a glioblastoma mouse model**

**Authors**

Masahiro Maeyama^1^ , Kazuhiro Tanaka^1^ , Masamitsu Nishihara^2^ , Yasuhiro Irino^3^ , Masakazu Shinohara^4,5^ , Hiroaki Nagashima^6^ , Hirotomo Tanaka^1^ , Satoshi Nakamizo^1^ , Mitsuru Hashiguchi^1^ , Yuichi Fujita^1^ , Masaaki Kohta^1^ , Eiji Kohmura^1^, Takashi Sasayama^1^

**Affiliations**

^1^Department of Neurosurgery, Kobe University Graduate School of Medicine, 7-5-1, Kusunoki-cho, Chuo-ku, Kobe 650-0017, Japan

^2^Department of Neurosurgery, Nishi-Kobe Medical Center, Kobe, Japan

^3^Division of Evidence-Based Laboratory Medicine, Kobe University Graduate School of Medicine

^4^Integrated Center for Mass Spectrometry, Kobe University Graduate School of Medicine

^5^Division of Epidemiology, Kobe University Graduate School of Medicine

^6^Department of Neurosurgery, Massachusetts General Hospital Research Institute, Boston, Massachusetts, USA.

**Supplemental Table S1**

Metabolome analysis of tumor tissue.

**Supplemental Table S2**

Metabolome analysis of normal brain tissue.

**Supplemental Table S3**

The results of DNA microarray analysis of each groups.

**Supplemental Table S4**

Dietary components of CLEA Rodent Diet CL-2 and KetoCal 4:1.

**Supplemental figure S1**


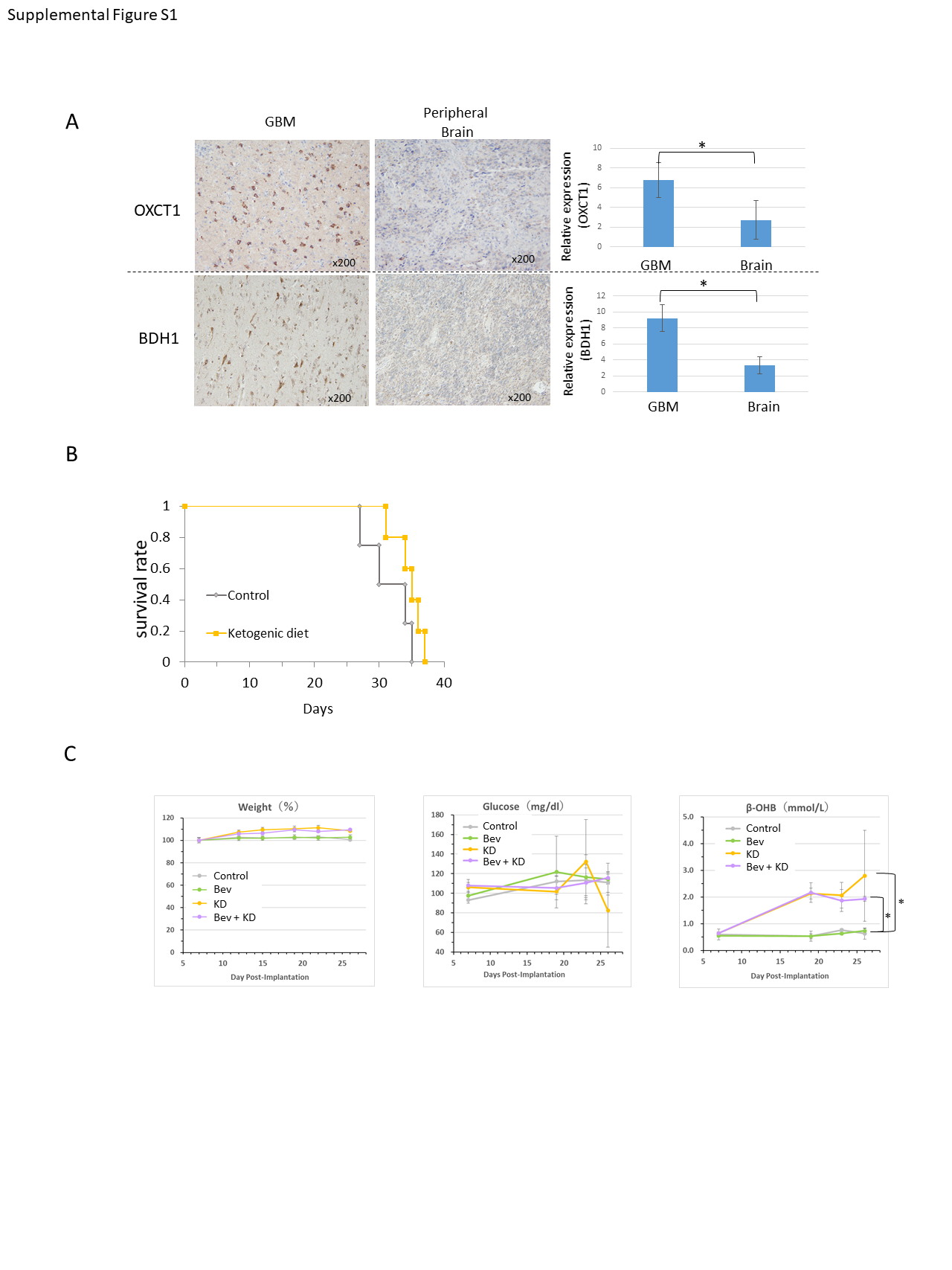


A: Immunostaining of ketone metabolizing enzymes (OXCT1 and BDH1) in brain and GBM. An intensity of immunostainings is quantified using image J software. B: Survival analysis of mice on a ketogenic diet. U87 cells are brain-transplanted into mice fed a normal diet (control) or a ketone diet and analyze for survival time. There is no significant difference between the two groups (p=0.32). C: Changes in body weight, blood glucose, and blood ketone body concentration in each group during the course of treatment. (*p<0.05)

**Supplemental figure S2**


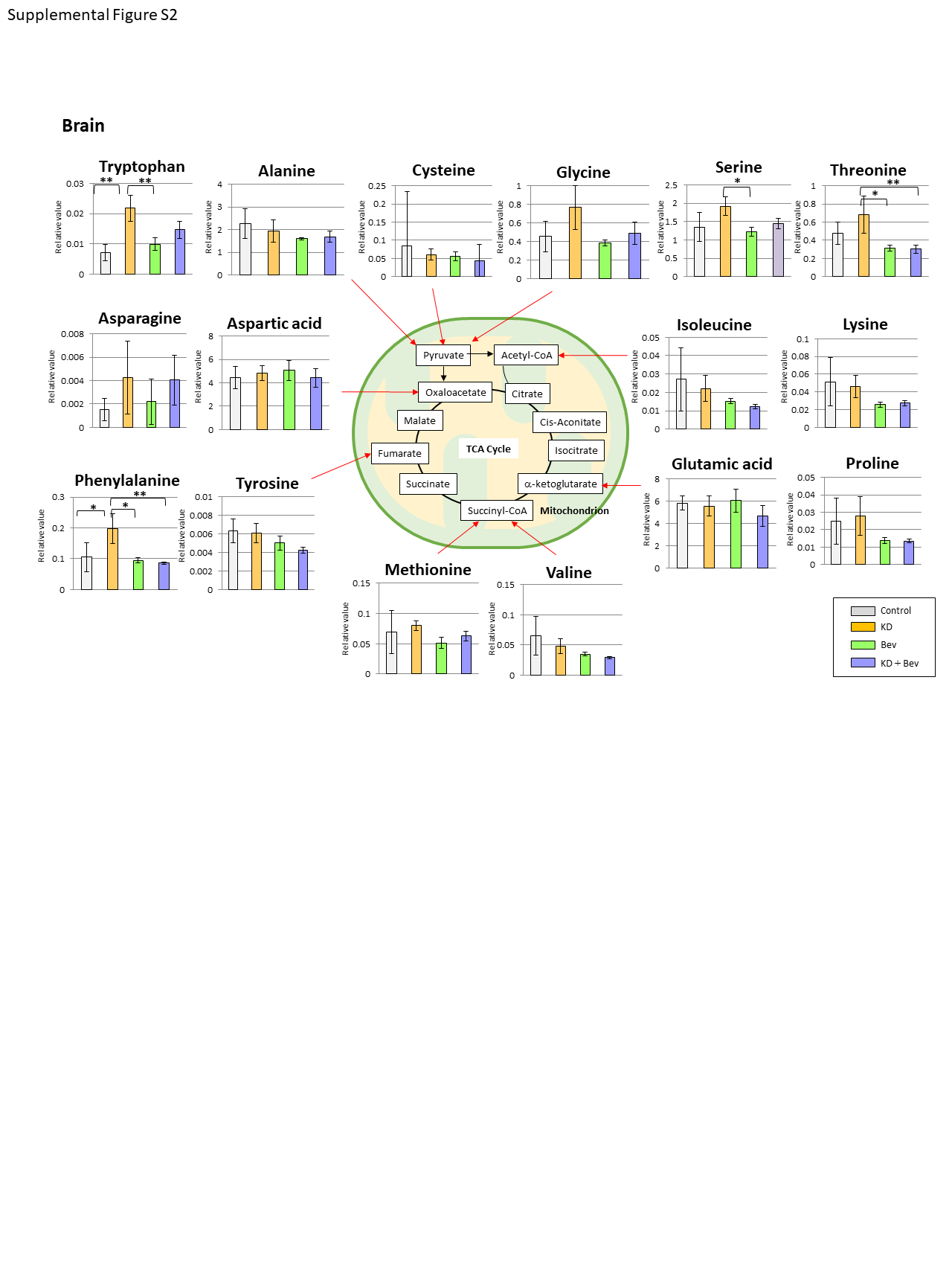


Comparison of the amino acids levels in the control, ketogenic diet (KD), bevacizumab (Bev), and ketogenic diet plus bevacizumab (KD+Bev) groups in normal bran tissues. Aspartic acid and glutamic acid are most different from the tumor. (*p<0.05, **p<0.01, and Tukey-Kramer test).

**Supplemental figure S3**


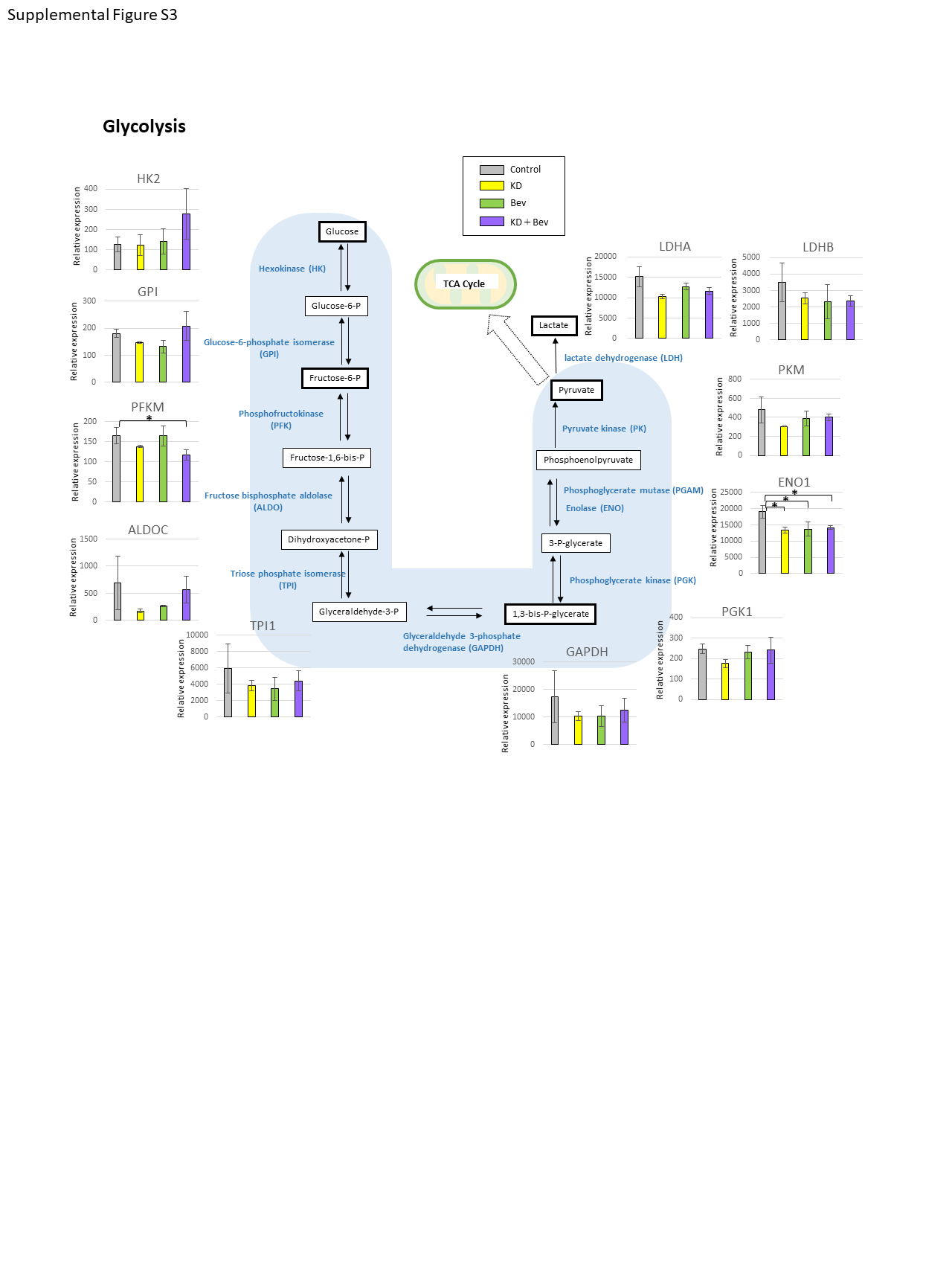


Comparison of mRNA expression levels of glycolytic enzymes (*p<0.05, **p<0.01, and Tukey-Kramer test).
